# miR-183-3p suppresses proliferation and migration of keratinocyte in psoriasis by inhibiting GAB1

**DOI:** 10.1186/s41065-020-00138-w

**Published:** 2020-07-10

**Authors:** Ting Liu, Xiaoyan Zhang, Yujuan Wang

**Affiliations:** Department of Dermatology, Affiliated Hospital of North Sichuan Medical College, No. 1, Maoyuan South Road, Nanchong City, 637000 Sichuan Province China

**Keywords:** miR-183-3p, GAB1, Psoriasis, Keratinocytes, Proliferation, Migration

## Abstract

**Background:**

MicroRNAs (miRNAs) target genes involved in the hyperproliferation of keratinocytes or immune dysfunction of psoriasis. This study prospectively determined the involvement of miR-183-3p in the pathogenesis of psoriasis.

**Methods:**

Differentially expressed miR-183-3p between psoriatic lesional and non-lesional skin were determined by quantitative RT-PCR and in situ hybridization (ISH). CCK8 and wound healing assays were performed to assess cell viability and migration of human keratinocyte cell line (HaCaT). The target of miR-183-3p was validated by luciferase activity assay.

**Results:**

Lower miR-183-3p expression was observed in psoriatic lesional skin compared to psoriatic non-lesional skin. MiR-183-3p over-expression inhibited the viability and migration of HaCaT cells, while inhibition of miR-183-3p promoted the viability and migration of HaCaT cells. Moreover, miR-183-3p could bind to the 3′ UTR of GAB1 (growth factor receptor binding 2-associated binding protein 1) and decrease the mRNA and protein expression of GAB1 in HaCaT cells. In addition, higher GAB1 expression was observed in psoriatic lesional skin than psoriatic non-lesional skin.

**Conclusion:**

MiR-183-3p exhibited inhibition property in the proliferation and migration of HaCaT cells via down-regulation of GAB1, suggesting the potential therapeutic strategy for psoriasis.

## Background

Psoriasis is a common chronic inflammatory disease, with increasing incidence and a prevalence between 1 and 3% worldwide [1]. The etiology and pathogenesis of psoriasis is complicated and still unclear. Currently, it is believed that the pathogenesis of psoriasis mainly involves immune, genetic, psychological and environmental factors [2]. The main pathological changes of psoriasis are keratinocyte dysplasia, keratinosis, neovascularization and inflammatory cell infiltration [3]. Meanwhile, hyperproliferation and abnormal migration of keratinocytes, responsible for psoriasis lesional microenvironment, are critical features of psoriasis [4]. Considering the fact that psoriasis is easy to relapse and difficult to cure, exploringnew therapeutical targets involved in the proliferation and migration of keratinocytes is beneficial for the cure of psoriasis.

MiRNAs (microRNAs), with a length of about 18–25 nucleotides, are a class of non-coding single-stranded small RNA molecules that are highly conserved in evolution and ubiquitous in plants and animals [5]. MiRNAs account for 1–5% of the entire human genome and regulate the expression of 30% of protein-coding genes in human [5]. MiRNAs could bind to the 3’UTR of a specific target gene mRNA through base complementary pairing to degrade its target mRNA or inhibit its translation, thereby negatively regulate protein expression at post-transcriptional levels [5]. Moreover, miRNAs have the abilities to regulate cell proliferation and immune response, are critically implicated in the pathogenesis of immunological disorders, including psoriasis [6]. For example, Sonkoly et al. found that miR-203 could inhibit the expression of suppressor of cytokine signaling-3, which was involved in the inflammatory response of keratinocyte [7]. MiR-125b reduced the expression of fibroblast growth factor receptor 2 to regulate the proliferation and differentiation of keratinocytes [8]. MiR-4516 could reduce the proliferatory and migratory abilities of keratinocytes toameliorate psoriasis [9].

MiR-183-3p was found to be associated with chronic systolic heart failure [10] and lung adenocarcinoma [11]. More recently, miR-183-5p was identified as a mediator of inflammatory disease and involved in the chronic constriction injury-induced neuropathic pain [12],. Besides, down-regulation of miR-183-3p was also discovered in psoriatic skin [13]. However, the role and mechanism of miR-183-3p in psoriasis remains elusive. Therefore, this study aimed to examine the functional role of miR-183-3p in the proliferation and migration of keratinocytes, and identified the underlying mechanism, thus providing more potential therapeutic strategy for psoriasis.

## Results

### MiR-183-3p was down-regulated in psoriatic lesional skin

**Fig. 1:**
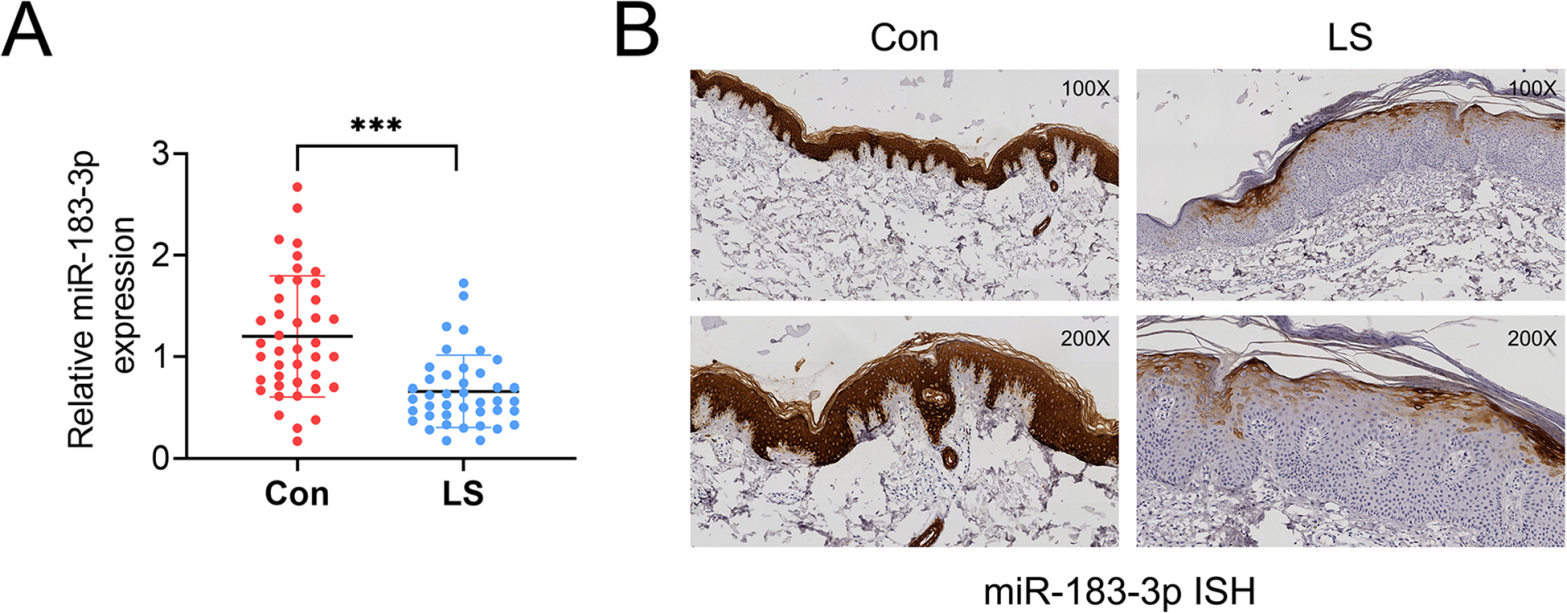
MiR-183-3p was down-regulated in psoriatic lesional skin. **a** MiR-183-3p was down-regulated in psoriatic lesional (LS) compared to non-lesional (Con) skin biopsies, examined by qRT-PCR. *** represents LS vs. Con, *p* < 0.001. **b** MiR-183-3p was down-regulated in psoriatic lesional (LS) compared to non-lesional (Con) skin biopsies, examined by ISH. Magnification, 100 X, 200 X

To evaluate the expression level of miR-183-3p in psoriatic patients, 41 paired psoriatic lesional (LS) or non-lesional (Con) skin biopsies were collected and qRT-PCR was peformed. Result showed a significant reduction of miR-183-3p in LS compared to Con (Fig. 1a). Moreover, ISH showed that higher expression of miR-183-3p in Con group was found in basal and suprabasal cell layers than that in LS group (Fig. 1b). In general, miR-183-3p was down-regulated in psoriatic lesional skin.

### Over-expression of miR-183-3p suppressed keratinocytes cell proliferation and migration

**Fig. 2:**
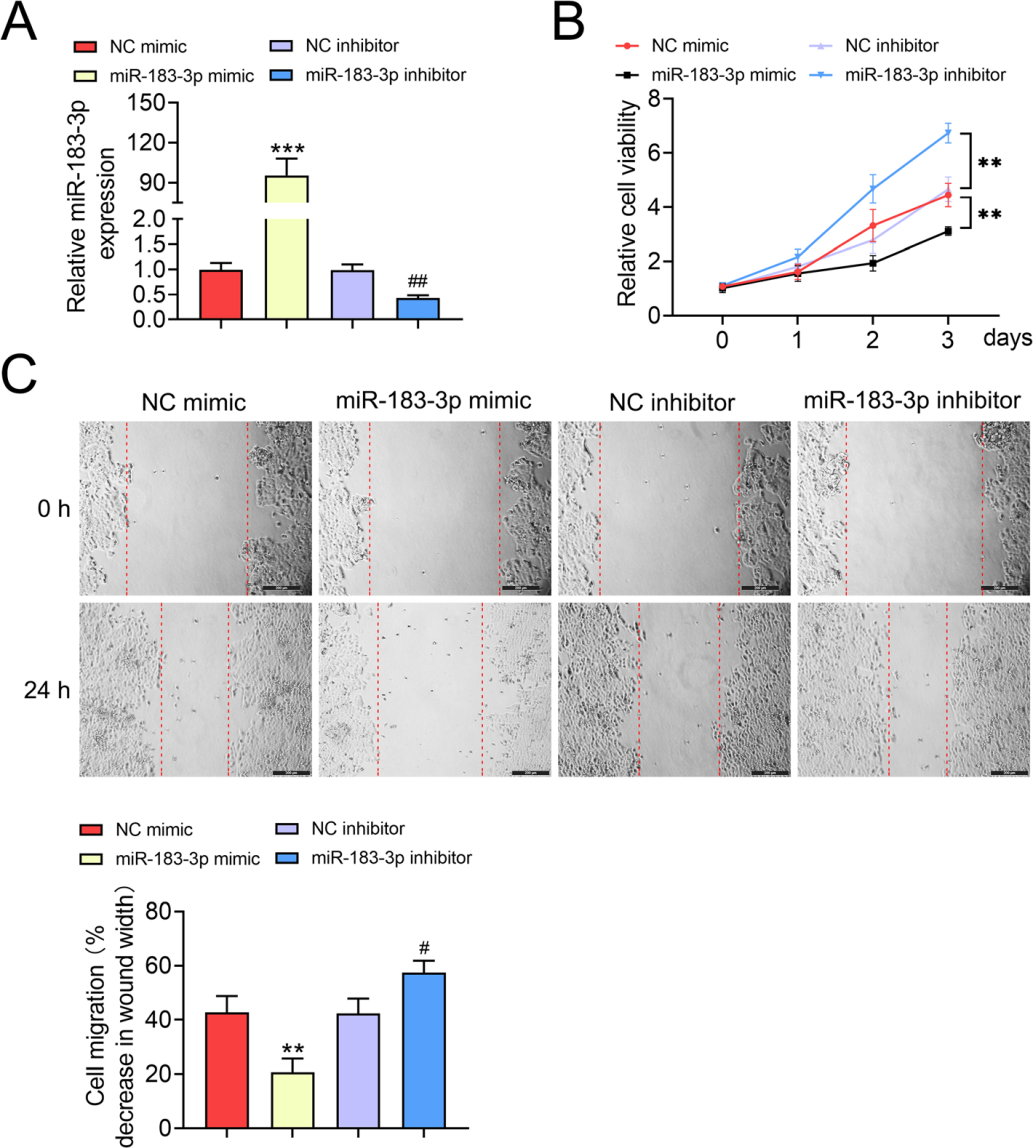
Over-expression of miR-183-3p suppressed keratinocytes cell proliferation and migration. **a** Transfection efficiency of miR-183-3p mimics or inhibitor in HaCaT cells. *** represents miR-183-3p mimics vs. NC mimic, *p* < 0.001. ## represents miR-183-3p inhibitor vs. NC inhibitor, *p* < 0.01. **b** MiR-183-3p decreased the viability of HaCaT cells, while miR-183-3p inhibitor increased the viability of HaCaT cells. ** represents miR-183-3p mimics vs. NC mimic, *p* < 0.01. ## represents miR-183-3p inhibitor vs. NC inhibitor, *p* < 0.01. **c** MiR-183-3p inhibited the migration of HaCaT cells, while miR-183-3p inhibitor promoted the migration of HaCaT cells. ** represents miR-183-3p mimics vs. NC mimic, *p* < 0.01. # represents miR-183-3p inhibitor vs. NC inhibitor, *p* < 0.05

To evaluate the biological role of miR-183-3p in keratinocyte, HaCaT cells were transfected with miR-183-3p mimics or inhibitor, and the transfection efficiency was validated by qRT-PCR (Fig. 2a). CCK8 assay revealed that over-expression of miR-183-3p inhibited HaCaT cell viability (Fig. 2b), while inhibition of miR-183-3p promoted HaCaT cell viability (Fig. 2b). Moreover, the migration of HaCaT cells was inhibited by miR-183-3p mimics (Fig. 2c), while promoted by miR-183-3p inhibitor (Fig. 2c). Therefore, over-expression of miR-183-3p suppressed keratinocytes cell proliferation and migration.

### MiR-183-3p negatively regulated GAB1 expression in keratinocytes

**Fig. 3:**
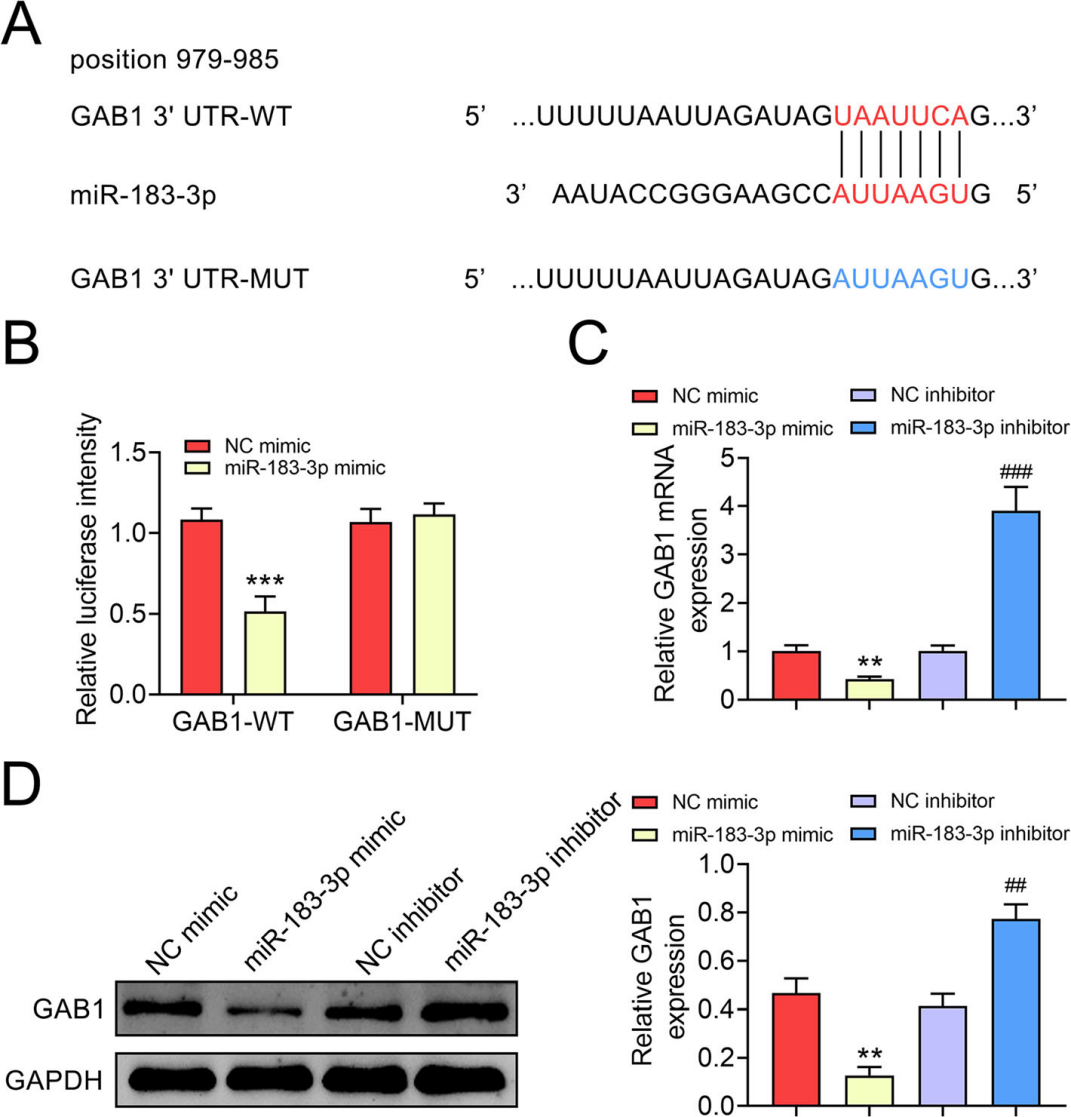
MiR-183-3p negatively regulated GAB1 expression in keratinocytes. **a** Putative binding sites between GAB1 and miR-183-3p. **b** MiR-183-3p decreased the luciferase activity of pmirGLO-wt-GAB1 compared to pmirGLO-mut-GAB1 in HaCaT cells. *** represents miR-183-3p mimics vs. NC mimic, *p* < 0.001. **c** MiR-183-3p decreased mRNA expression of GAB1 in HaCaT cells, while miR-183-3p inhibitor increased GAB1 expression. ** represents miR-183-3p mimics vs. NC mimic, *p* < 0.01. ### represents miR-183-3p inhibitor vs. NC inhibitor, *p* < 0.001. **d** MiR-183-3p decreased protein expression of GAB1 in HaCaT cells, while miR-183-3p inhibitor increased GAB1 expression. ** represents miR-183-3p mimics vs. NC mimic, *p* < 0.01. ## represents miR-183-3p inhibitor vs. NC inhibitor, *p* < 0.01

Thebinding site of miR-183-3p was predicted and results demonstrated that 3′ UTR of GAB1 harbored putative binding site for miR-183-3p (Fig. 3a). The dual luciferase assay indicated that ectopic expression of miR-183-3p repressed the luciferase activity of pmirGLO-wt-GAB1 (Fig. 3b), while this inhibitory effect was abolished by transfection with pmirGLO-mut-GAB1 (Fig. 3b). In addition, miR-183-3p mimics reduced the mRNA and protein expression of GAB1, while GAB1 expression was increased in HaCaT cells after transfected with miR-183-3p inhibitor (Fig. 3c and d). Taken together, miR-183-3p negatively regulated GAB1 expression in keratinocytes.

### GAB1 was up-regulated in psoriatic lesional skin

**Fig. 4:**
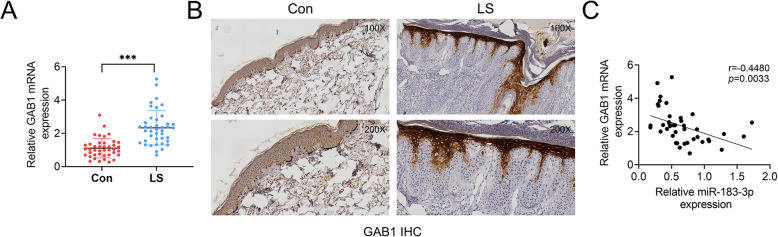
GAB1 was up-regulated in psoriatic lesional skin. **a** GAB1 was up-regulated in psoriatic lesional (LS) compared to non-lesional (Con) skin biopsies examined by qRT-PCR. *** represents LS vs. Con, *p* < 0.001. **b** GAB1 was up-regulated in psoriatic lesional (LS) compared to non-lesional (Con) skin biopsies examined by IHC. Magnification, 100 X, 200 X. **c** Negative correlation between miR-183-3p and GAB1 in psoriatic patients

Considering the negative corrlation between miR-183-3p and GAB1, the expression of GAB1 in psoriatic lesional skin was then determined. Results fromqRT-PCR (Fig. 4a) and immunohistochemistry (Fig. 4b) showed that GAB1 was elevated in LS group compared to Con group. Moreover, the correlation analysis indicated a significantly negative correlation (*p* = 0.0033) between miR-183-3p and GAB1 in psoriatic patients (Fig. 4c).

## Discussion

Increasing evidence has revealed that miRNAs were abnormally expressed in psoriasis, and regulate immune dysfunction and keratinocytes proliferation in psoriasis [6]. Results from this study revealed the downregulation of miR-183-3p in psoriatic lesional skin, which were consistent with a previous report [13], indicating its regulatory role in the progression of psoriasis.

Psoriasis is a complicated disease resulting from the dysregulated interplay between keratinocytes, immune cells and other skin-resident cells [14]. Hyperproliferation of keratinocytes could result in tacanthosis, infiltration of immunocytes in the epidermis that are resemble with psoriasis lesional microenvironment [15]. Moreover, the migration of keratinocytes could promote wound re-epithelialization and recapitulate psoriasis lesional microenvironment [16]. Inhibition of keratinocytes proliferation and migration could be beneficial for psoriasis [9]. This study also demonstrated that ectopic expression of miR-183-3p inhibited keratinocytes proliferation and migration, while inhibition of miR-183-3p promoted keratinocytes proliferation and migration. Moreover, keratinocytes are rekated to cell apoptosis in psoriasis [17], and inhibition of cell apoptosis contribute to the amelioration of psoriasis (MiR-20a-3p regulates TGF-β1/Survivin pathway to affect keratinocytes proliferation and apoptosis by targeting SFMBT1 in vitro). Therefore, the role of miR-183-3p in the apoptosis of keratinocytes should be investigated in the further study. In addition, psoriasis is also a chronic inflammatory disease [1], accumulation of proinflammatory factor can exacerbate psoriasis [18]. Numerous inflammatory cytokines secreted from immune cells could contribute to the proliferation of keratinocytes [19]. IL22, up-regulated in lesional skins of patients with psoriasis [20], could bind to the receptor in keratinocytes and promote the cell proliferation [21], thus is critical for the pathogenesis of psoriasis. Additionally, miR-183, abnormally expressed in autoimmune diseases, plays key role in immunity [22]. MiR-183c could suppress Th17 cell pathogenic function [23], which is also important for psoriasis. Further investigations should concernthe effect of miR-183-3p on inflammation during psoriasis.

Bcl-2 interacting protein 3 like [10] or high-mobility group nucleosome binding domain 5 [24] were validated as binding targets of miR-183-3p and involved in chronic systolic heart failure or prostate cancer, respectively. The direct binding target involved in miR-183-3p-mediated psoriasis remains elusive. This is the first study identifing GAB1 as a binding target of miR-183-3p during psoriasis. GAB1 functions as a docking protein to promote the proliferation and differentiation of epidermis [25]. Moreover, GAB1 was found to be involved in the migration of keratinocyte, as well as wound healing, suggesting its potential role in the pathogenesis of psoriasis [26]. Recently, GAB1 was shown to be aberrantly expressed in multiple sclerosis and psoriasis [27]. Results from this study showed that GAB1 was up-regulated in psoriatic lesional skin, and suggested a significantly negative correlation with miR-183-3p. In addition, miR-183-3p could repress the mRNA and protein expression of GAB1, suggesting that miR-183-3p could suppress the proliferation and migration of keratinocyte in psoriasis by inhibiting GAB1. Ras [25] and phosphoinositide 3-kinase pathway [26] were closely associated with GAB1-mediated epidermal differentiation and cell proliferation/migration in cultured skin keratinocytes, respectively. The underlying mechanism related to miR-183-3p/GAB1 axis-mediated psoriasis needs further investigations.

## Conclusion

In conclusion, miR-183-3p effectively suppressed the viability and migration of keratinocytes through down-regulation of GAB1. These results suggested the beneficial effect of miR-183-3p knockdown during psoriasis.

## Methods

### Patients and specimens

Forty-one paired lesional or non-lesional skin biopsies (5 mm) were collected from patients who diagnosed as psoriasis at the Affiliated Hospital of North Sichuan Medical College. The protocol was approved by the Ethics Committee of Affiliated Hospital of North Sichuan Medical College, and all patients signed written informed consent.

### In situ hybridization

Formalin-fixed and paraffin-embedded sections (5 μm in thickness) of the biopsy specimens were firstly incubated with acetylation solution for 10 min, and then incubated with permeabilization buffer for 30 min. After prehybridizing at 50 °C for 1 h, the sections were hybridized with digoxygenin-labeled miRCURY locked nucleic acid probes (Exiqon, Vedbaek, Denmark) overnight. After incubation with alkaline phophatase–conjugated sheep antidigoxigenin Fab fragments (1:3000, Abcam, Cambridge, MA, USA) for 1 h, the sections were visualized under inverted microscope (Nikon Eclipse Ti, Tokyo, Japan).

### Immunohistochemistry

Formalin-fixed and paraffin-embedded sections (0.5 μm in thickness) of the biopsy specimens were incubated with rabbit antihuman GAB1 antibody (1:500, Abcam) overnight. After incubation with horse radish peroxidase-goat anti-rabbit secondary antibody, the sections were visualized under inverted microscope.

### Cell culture

HaCaT cells were cultured in 37 °C constant temperature incubator with 5% CO_2_ in DMEM F-12 media (Life Technologies, Gaithersburg, MD, USA) with 10% fetal bovine serum (Gibco, Grand Island, NY, USA) and streptomycin (100 μg/ml) and penicillin (100 U/ml).

### Cell transfection

MiR-183-3p mimics, inhibitor and the negative controls (NC mimic, NC inhibitor) were synthesized by GenePharma (Suzhou, China). HaCaT cells (1 × 10^6^ per well) were seeded and then transfected with miR-183-3p mimics/inhibitor (20 nM) or their negative controls (NC) via Lipofectamine 2000. The transfection efficiency was validated by qRT-PCR 48 h after transfection.

### Cell viability

HaCaT cells (1 × 10^4^ cells/well) were seeded and then transfected with miR-183-3p mimics/inhibitor (20 nM) or their NC. Forty-eight hours later, each well was supplemented with 10 μL CCK8 solution (Dojindo, Tokyo, Japan) for 1 h. Absorbance at 450 nm for each well was determined via Microplate Autoreader (Thermo Fisher, Waltham, MA, USA) every 24 h intervals at 0, 24, 48, 72 h.

### Wound healing

HaCaT cells (1 × 10^4^ cells/well) were seeded and then transfected with miR-183-3p mimics/inhibitor (20 nM) or their NC. After transfection for 48 h, each well was scratched and then cultured for another 24 h. Wound area was photographed and calculated.

### Dual luciferase reporter assay

Sequences of wildtype or mutant of GAB1 3′-UTR were constructed into pmirGLO luciferase reporter vector (Promega, Madison, Wisconsin, USA). The seeded HaCaT cells were co-transfected with miR-183-3p mimics or NC mimic and pmirGLO-wt-GAB1 or pmirGLO-mut-GAB1. Two days later, the luciferase activities were determined via Lucifer Reporter Assay System (Promega).

### qRT-PCR

RNAs or miRNAs were isolated from psoriatic lesional or non-lesional skin and HaCaT cells via Trizol (Thermo Fisher). Total RNAs or miRNAs were then reverse-transcribed into cDNAs via High Capacity Reverse Transcription System Kit (Takara, Dalian, China) or MicroRNA Reverse Transcription Synthesis Kit (Thermo Fisher). qRT-PCR was conducted with SYBR Green Master (Roche, Mannheim, Germany) on Applied Biosystems 7500 Real-time PCR Systems (Thermo Fisher). GAPDH or U6 was used as endogenous control with the following primer sequences (Table 1).

**Table 1:** Primer

ID	Sequence (5’- 3’)
GAPDH F	TATGATGATATCAAGAGGGTAGT
GAPDH R	TGTATCCAAACTCATTGTCATAC
miR-183-3p F	CGCGGTATGGCACTGGTAGA
miR-183-3p R	AGTGCAGGGTCCGAGGTATTC
GAB1 F	ATGAGCGGCGGCGAAGTGGTTTGCT
GAB1 R	CGCGACTGAAGAAGCTTCCATCTGA
U6 F	CTCGCTTCGGCAGCACA
U6 R	AACGCTTCACGAATTTGCGT

### Western blot

Proteins were extracted from HaCaT cells and subjected to SDS-PAGE. After transferring onto PVDF membrane and blocking by 5% BSA, the membranes were then incubated with primary antibodies: anti-GAB1 (1:1500, Abcam) and GAPDH (1:3000, Abcam), followed by incubulation with HRP labeled secondary antibody (Sungene, Tianjin, China). The immunoreactivities were determined by Millipore ECL (Billerica, MA, USA).

### Statistical analysis

Data were shown as mean ± SEM and analyzed by Graphpad Prism 6. The statistical analyses were determined with one-way analysis of variance at significance of * *p* < 0.05, ** *p* < 0.01 or *** *p* < 0.001. The correlation between GAB1 and miR-183-3p expression was determined by Spearman’s correlation coefficients.

## Data Availability

All data generated or analyzed during this study are included in this published article.

## Abbreviations

miRNAs: MicroRNAs
ISH: In situ hybridization
HaCaT: Human keratinocyte cell line
